# Hydrothermal Synthesis of Various Magnetic Properties of Controlled Micro/Nanostructured Powders and Films of Rare-Earth Iron Garnet

**DOI:** 10.3390/nano11040972

**Published:** 2021-04-10

**Authors:** Natalia Tsidaeva, Ahsarbek Nakusov, Spartak Khaimanov, Wei Wang

**Affiliations:** 1 Scientific Center “Magnetic Nanostructures”, North Caucasus Mining and Metallurgical Institute, State Technological University, 44, Nikolaeva str., 362021 Vladikavkaz, Russia; shasha_nat@mail.ru (A.N.); sh_khaymanov@mail.ru (S.K.); 2 Department of Physics and Electronics, School of Mathematics and Physics, Beijing University of Chemical Technology, Beijing 100029, China; wangwei@mail.buct.edu.cn; 3 Beijing Key Laboratory of Environmentally Harmful Chemical Analysis, Beijing University of Chemical Technology, Beijing 100029, China

**Keywords:** rare-earth iron garnets, micro/nanostructured powders and films, X-ray diffraction analysis, atomic force microscopy, Raman spectroscopy, surface topology, magnetic properties

## Abstract

In this study, the synthesis and magnetic properties of the rare-earth iron garnets Sm_3_Fe_5_O_12_, Pr_3_Fe_5_O_12_, and Er_3_Fe_5_O_12_ (in the form of powders and thin films) are reported. According to the composition, shape, and size of particles, the optimal precipitant for the synthesis of Sm_3_Fe_5_O_12_, Pr_3_Fe_5_O_12_, and Er_3_Fe_5_O_12_ films is an aqueous solution. The parameters for the synthesis of powders and films of the rare-earth iron garnets with micro- and nano-particles have been investigated and selected. The magnetic properties of these materials were studied; field dependencies of the magnetic moment (hysteresis loops) of nanostructured powders of iron garnets of samarium, praseodymium, and erbium in the range of +20 kOe to −20 kOe were obtained. The structural features of the Al_2_O_3_ substrate on which the films were formed are also shown.

## 1. Introduction

The study of materials based on iron garnets has long been a research focus for scientists and research on this topic continues to develop rapidly as a result of the increasing scope of these materials [1,2,3,4]. Such materials are attractive due to their unique magnetic, optical, sensory, and catalytic properties [5,6,7,8,9]. The use of nanosized samples based on these materials has revealed radically new properties and led to increased interest in these objects from both a fundamental and practical point of view., In particular, related to iron garnets, which have been successfully used in various fields of modern science and technology, as well as in medicine and biochemistry. The ability to control movement using an external constant magnetic field is one of the advantages of iron garnet nanoparticles.

The greatest degree of success in the study of the properties and parameters of rare-earth iron garnets has been achieved using modern equipment and advanced methods of physical and chemical analysis [10,11,12,13,14,15].

Nevertheless, the studies of various authors and scientific groups that are being carried out at present have a number of drawbacks, including the expense of methods required to obtain samples, the synthesis of materials with low magnetic and adsorption properties, and the use of bulk materials (single crystals, films with a thickness of more than 100 μm) [16,17,18].

The main goal of this work is to determine regularities in the series ‘synthesis method, composition, structure, properties’ using examples of nanostructured powders and films of Sm_3_Fe_5_O_12_, Pr_3_Fe_5_O_12_, and Er_3_Fe_5_O_12_, the formation stages of which are an important component of the manufacture of functional materials.

To achieve this goal, it was necessary to solve the following tasks:

− examine the possibility of using the presented synthesis methods to form powders and films of Sm_3_Fe_5_O_12_, Pr_3_Fe_5_O_12_, and Er_3_Fe_5_O_12_;
− determine the possibility of using Al_2_O_3_ substrate for the growth of the Sm_3_Fe_5_O_12_, Pr_3_Fe_5_O_12_, and Er_3_Fe_5_O_12_ films;
− compare the elemental composition of the obtained powders and films, as well as the substrate material used, to the composition of reference samples;
− determine the chemical composition and the degree of structural perfection of the powders and films formed by methods of X-ray diffraction analysis, Raman spectroscopy, and atomic force microscopy.

## 2. Materials and Methods

Various methods can be used for the production of rare-earth iron garnets. Their diversity is explained by the difference in the chemical nature of the starting materials and substrate material, the different purposes for which they are made, and the various conditions of use.

To solve the problems outlined in the Introduction, it is necessary to select the optimal methods from those synthesized that contribute to obtaining the required structural, electrophysical and magnetic parameters.

The synthesis of powders was carried out using the hydrothermal method. The advantages, parameters and stages of this method were previously described in detail by us and the authors of other works [19,20,21,22,23].

It should be noted that through the course of the experiment, we used samples with an alkali concentration (NaOH) of 14 g per 50 mL of the initial solution, and the solution was processed at 240 °C. We maintained the same synthesis parameters to show how different chemical compositions affected the properties of the materials under study.

The synthesis method of the films studied in this work can be classified as hydrochemical deposition of aqueous solutions on a substrate surface. Evaluating the advantages of this method in comparison with others, one can single out its high productivity, simplicity of technological processes, and cost-effectiveness.

The synthesis of the films was carried out in two stages:

(1) Synthesis of the Sm_3_Fe_5_O_12_, Pr_3_Fe_5_O_12_ and Er_3_Fe_5_O_12_ powders via the hydrothermal method.
(2) Formation of the films by hydrochemical deposition of aqueous solutions on the surface of the substrate material. Specifically, the synthesis of the samples was carried out by applying aqueous solutions of Sm_3_Fe_5_O_12_, Pr_3_Fe_5_O_12_ and Er_3_Fe_5_O_12_ + H_2_O on the surface of an Al_2_O_3_-based substrate. To improve the adhesion of the film and the substrate material and to remove the residual reaction products from the solution, the samples were subjected to heat treatment at 80 °C for 1 h at a pressure of 0.07 MPa.

The initial reagents used for the synthesis of the powders and films are presented in Table 1.

All of the chemicals purchased from Sinopharm Chemical Reagent Company Ltd., Shanghai, China, were of analytical grade and were not processed further.

**Figure 1:**
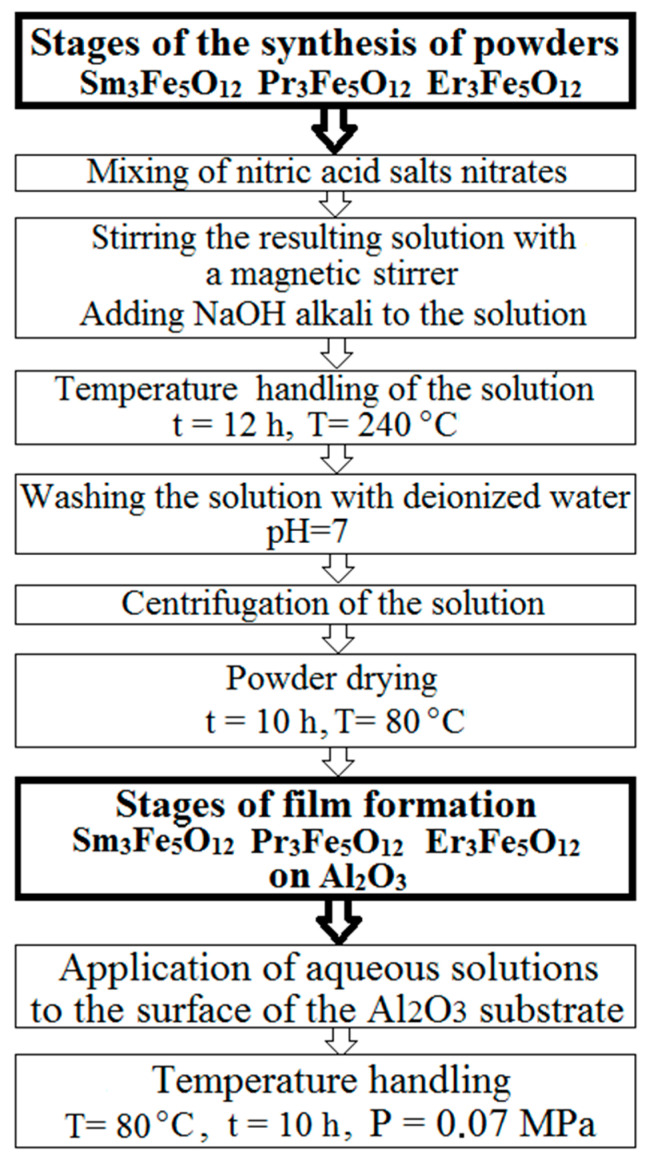
Stages of synthesis of nanostructured powders and films of Sm_3_Fe_5_O_12_, Pr_3_Fe_5_O_12_, and Er_3_Fe_5_O_12_.

The chemical reactions that occurred during the synthesis are described in detail in [22]. A schematic representation of the synthesis stages of the samples under study is shown in Figure 1.

The choice of the temperature regime and the amount of alkali in the synthesis were determined by the optimal properties of the samples, which were confirmed by the results of previous studies described in the work [22].

### Research Methods

**Figure 2:**
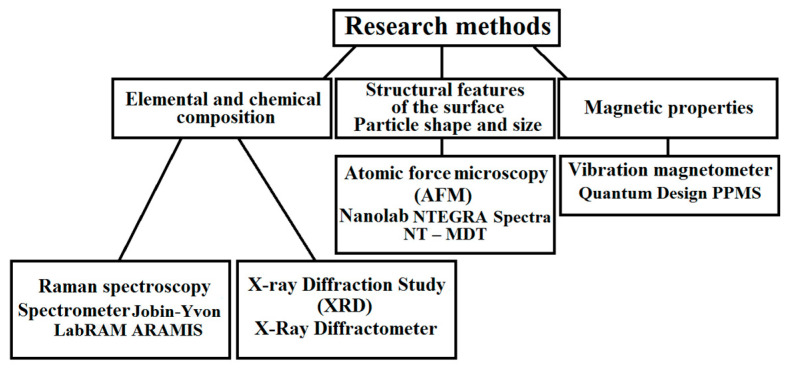
Methods used to analyze the samples and equipment used in this work.

The properties and parameters of the samples were obtained using various analytical methods. In general, these methods can be divided into two groups: Physical and chemical methods, and electron microscopic methods. Figure 2 shows the methods and equipment that were used in the research, as well as the parameters studied. The use of these techniques made it possible to establish the structure and functional properties of the powders and films, as well as the characteristics of the substrate materials used.

The equipment and parameters of the research methods will be described in more detail in the third part of this paper.

## 3. Results

### 3.1. Elemental and Chemical Composition

**Figure 3:**
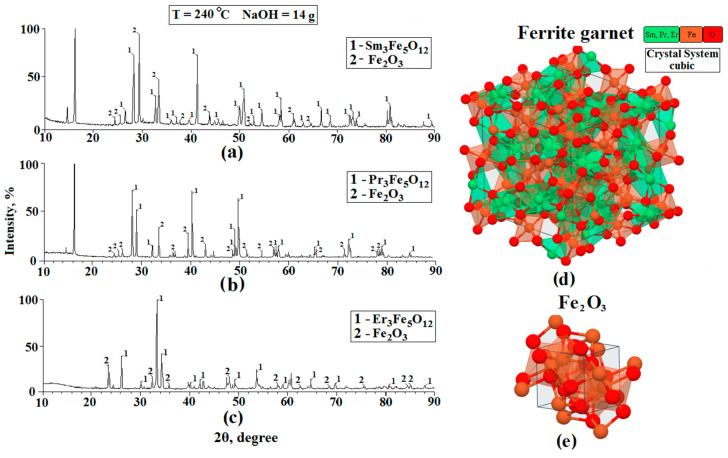
X-ray diffractograms of the Sm_3_Fe_5_O_12_ (**a**), Er_3_Fe_5_O_12_ (**b**), and Pr_3_Fe_5_O_12_ (**c**) samples and also the image of the structure of ferrite garnets (**d**) and structure Fe_2_O_3_ (**e**).

The elemental and chemical composition of the synthesized powders were studied by X-ray diffraction (XRD) and Raman spectroscopy. Sm_3_Fe_5_O_12_, Pr_3_Fe_5_O_12_, and Er_3_Fe_5_O_12_ powders were used as the objects of the study. In the case of XRD, the experiment was carried out using a Bruker DS Advance diffractometer equipped with a vertical goniometer using monochromatic copper radiation with a wavelength of CuKα = 1.54 Å with a 2θ range of 10–90° (during the study, both the X-ray tube and the receiver moved). The phases and crystal structure were identified using the intensity ratios and angles of the characteristic peaks in the diffraction patterns. We used the publicly available X-ray diffractogram databases (Match 2 and DASH 3.3.6) to compare and confirm the results obtained. The results of the XRD analysis of the powders based on Sm_3_Fe_5_O_12_, Pr_3_Fe_5_O_12_, and Er_3_Fe_5_O_12_ are shown in Figure 3.

Based on the data obtained, we can conclude that:

− The diffraction patterns of the films and the substrate showed peaks corresponding to the cubic structures of iron garnets of samarium, erbium, and praseodymium;
− The peaks corresponding to the individual elements included in the composition of ferrite-garnet were not observed in the diffractograms, i.e., according to the XRD, the samples corresponded to Sm_3_Fe_5_O_12_, Pr_3_Fe_5_O_12_, and Er_3_Fe_5_O_12_;
− In addition to the peaks corresponding to the studied ferrite-garnets, Fe_2_O_3_ peaks are observed on the diffractograms. This material is a residual reaction product formed during the synthesis. The appearance of iron oxide is due to the fact that a small part of the ferrite-garnet is not fully formed. This statement is also confirmed in the work [24].
− The chemical composition of the studied samples was confirmed by comparing the experimental diffraction patterns with results published in publicly available databases (Match 2 and DASH 3.3.6).
− On the diffractograms Sm_3_Fe_5_O_12_ and Pr_3_Fe_5_O_12_, peaks located at 17° are observed. These peaks cannot be identified, since they do not correspond to any material that is formed at the final stages of hydrothermal synthesis. In our opinion, this peak corresponds to one of the impurity materials formed during the intermediate stages of the synthesis of ferrites-garnets. This material is a residual product of the reaction.
− We do not exclude the possibility of formation of such materials as samarium orthoferrite, praseodymium orthoferrite, erbium orthoferrite, and erbium oxide during the synthesis process.

**Figure 4:**
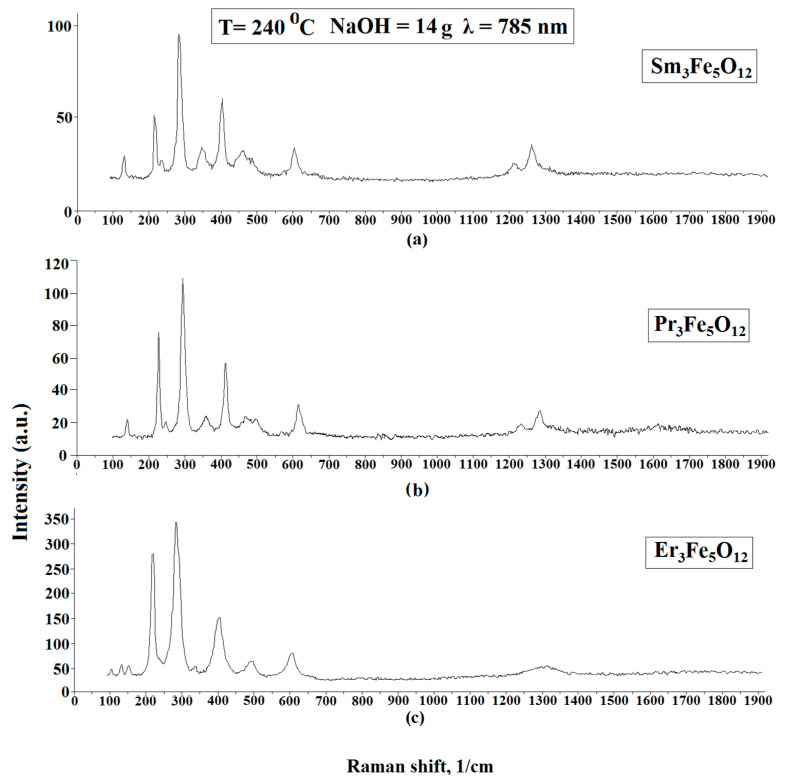
Raman spectra of Sm_3_Fe_5_O_12_ (**a**), Pr_3_Fe_5_O_12_ (**b**), Er_3_Fe_5_O_12_ (**c**) powder samples.

Dispersive Raman spectroscopy was also used to study the materials. Raman spectroscopy is used to observe inelastically scattered light, enabling the identification of vibration states (phonons) of molecules. This method facilitates the identification of chemical elements that have a similar molecular structure, as their Raman spectrum is sufficiently different. Also, as in the case of XRD, powders were used as objects of research. The results of the Raman spectroscopy are shown in Figure 4. The measurements at various points in the samples showed that the structure was identical at all points studied. The peaks of the Raman spectra indicated the presence of Sm_3_Fe_5_O_12_, Pr_3_Fe_5_O_12_, and Er_3_Fe_5_O_12_. The study was carried out at room temperature and at different points, the intensity of the spectra changed slightly. The laser radiation source used for atomic excitation was a diode semiconductor with a wavelength of 785 nm and a power of 35 mV. A comparison of the obtained spectra with reference data [25,26] and the results reported in [27] confirmed that the samples had been successfully prepared. The spectra of Sm_3_Fe_5_O_12_, Pr_3_Fe_5_O_12_, and Er_3_Fe_5_O_12_ are very similar due to their isomorphic structure. The main Raman-active modes of garnets are 3A1g + 8Eg + 14F2g, and the Raman peaks are reasonably and qualitatively attributed to internal, translational, and rotational modes, respectively [28,29].

### 3.2. Surface Structural Features—Particle Size and Shape

**Figure 5:**
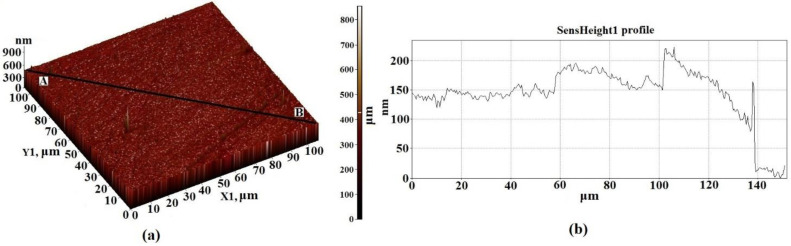
AFM 3D—image of the surface topology of a 100 × 100 μm^2^ section of substrate based on Al_2_O_3_ (**a**), and the height difference in the direction of the segment A–B (**b**).

**Figure 6:**
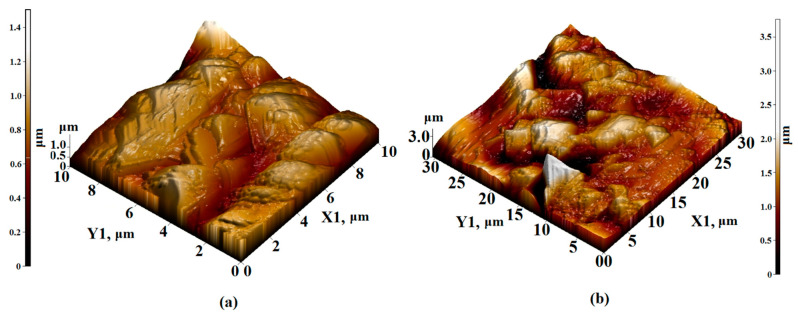
AFM 3D—image of the surface topology of (**a**) 10 × 10 µm^2^ and (**b**) 30 × 30 µm^2^ sections of the Sm_3_Fe_5_O_12_ film formed on an Al_2_O_3_ substrate.

**Figure 7:**
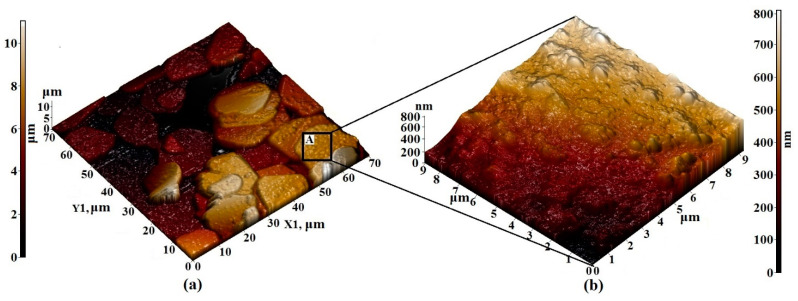
AFM 3D—image of the surface topology of (**a**) 70 × 70 µm^2^ and (**b**) 9 × 9 µm^2^ sections of the Pr_3_Fe_5_O_12_ film formed on an Al_2_O_3_ substrate.

**Figure 8:**
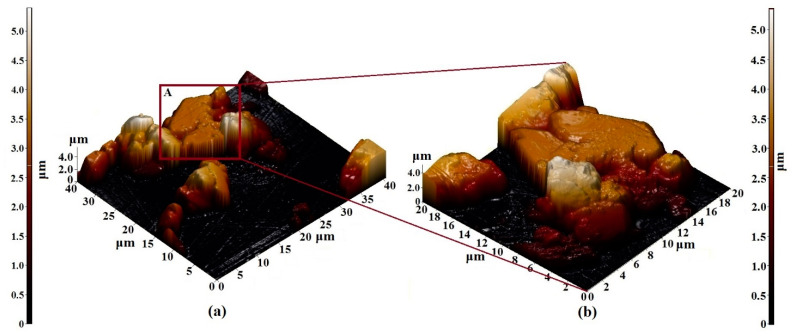
AFM 3D—image of the surface topology of (**a**) 40 × 40 µm^2^ and (**b**) 20 × 20 µm^2^ sections of the Er_3_Fe_5_O_12_ film formed on the Al_2_O_3_ substrate.

The study of the surface topology and shape and size of the particles of the obtained samples was carried out using atomic force microscopy (AFM). The films formed on the Al_2_O_3_ substrate were used as the samples. The results of studying the Sm_3_Fe_5_O_12_ powders obtained with identical synthesis parameters are presented in another work [22]. It was important for the test that the surface of the samples was sufficiently flat; this is necessary due to the technological limitations of the AFM method; namely, surface irregularities (large and sharp changes in heights of more than 10 μm) knock out the oscillating cantilever from the operating mode, which diminishes the quality of the experiment. The experimental data obtained by AFM are shown in Figure 5, Figure 6, Figure 7 and Figure 8.

The structure of the Al_2_O_3_ substrate material as the investigating methods of magnetic properties of the thin film structures were reported in our previous studies [22,30,31]. It should be noted that when scanning the maximum possible area of 100 × 100 μm^2^, the surface consists of particles with sizes of 50–600 nm and height differences of 800 nm (Figure 5). The surface irregularities of Al_2_O_3_ are rather high and the height difference was reduced by preliminary processing of the substrate surface (grinding). The obtained parameters of the substrate were generally satisfactory for their use in the experiment described here. The results of the study of films formed on a substrate using the combined AFM + Raman method are also presented in [22].

When scanning the sections of the Sm_3_Fe_5_O_12_ film of 10 × 10 μm^2^ and 30 × 30 μm^2^, agglomerates without a definite orientation were visible, with sizes varying from 1 to 1.5 μm. The height of the agglomerates varied from 0.8 to 1.2 microns. Clearly defined edges were observed and the surface topology was not oriented (Figure 6).

The visualization of small details of the surface layers of Pr_3_Fe_5_O_12_ films allows us to note the regularity in which the primary particles forming the film were combined into agglomerates with a rounded shape and diameters varying from 10 to 20 μm (Figure 7a). The size of primary particles varied from 100 to 500 nm (Figure 7b). Due to the attraction and subsequent overlapping of agglomerates, the maximum height difference of the synthesized films was quite high, amounting to 10 μm over the entire area of the scanned surface.

The results of the study of the structural features of the Er_3_Fe_5_O_12_ film by the AFM method are shown in Figure 8. Based on the images obtained, the following can be noted:

− The film was formed from particles with a diameter varying from 5 to 10 microns;
− The particle length was about 3 microns;
− The particles were rectangular (longer in the horizontal direction);
− The use of samples with such particle sizes as adsorption materials or materials for the manufacture of magnetic sensors is not possible. This is because a decrease in particle size leads to an increase in the magnetic, adsorption, and sensor properties of materials;
− It is necessary to select synthesis parameters that would reduce the particle size of the Er_3_Fe_5_O_12_ samples.

### 3.3. Magnetic Properties

The magnetic properties of the iron garnet are related to their microstructure, morphology, particle size, and composition. As the properties of the materials depend on the interatomic distances, it can be assumed that the saturation magnetization (Ms), Curie temperature (Tc), and other parameters related to the ferromagnetic state of nanostructures will differ from the analogous characteristics of bulk materials.

**Figure 9:**
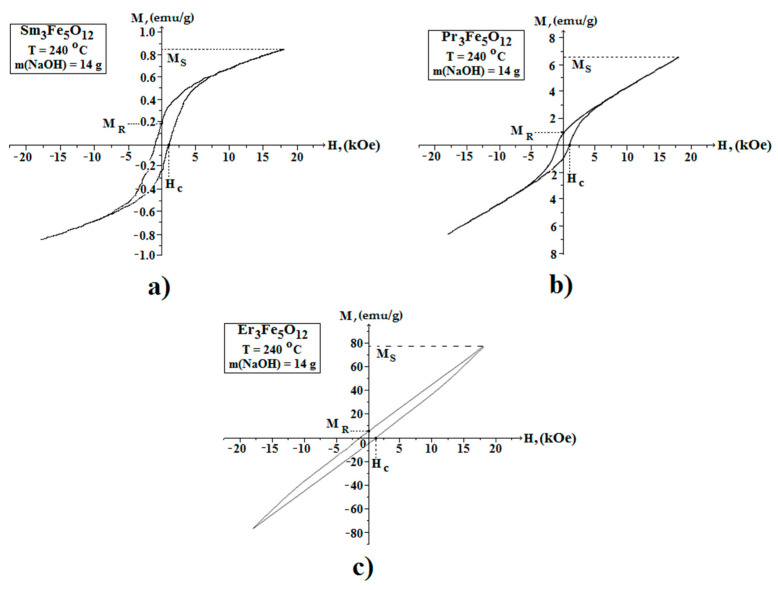
Magnetic hysteresis loops of the Sm_3_Fe_5_O_12_ (**a**), Pr_3_Fe_5_O_12_ (**b**), and Er_3_Fe_5_O_12_ (**c**) powder samples. Magnetic experiments were carried out at room temperature.

In the course of this work, we studied the field dependences of the magnetic moment (hysteresis loop) of the nanostructured powders of iron garnets of samarium, praseodymium, and erbium in the range of +20 kOe to −20 kOe. Figure 9 shows the magnetic hysteresis loops (M–H) of the Sm_3_Fe_5_O_12_, Pr_3_Fe_5_O_12_, and Er_3_Fe_5_O_12_ samples. The saturation magnetization (Ms) values were obtained from the magnetization curves simply by considering the largest possible values of the magnetization 0.85, 6.6, and 78 emu/g, respectively, recorded at the highest applied magnetic field of 20 kOe. Based on the obtained hysteresis loops, it should be noted that the highest remnant magnetization, coercive force, and saturation magnetization in the series of the materials under study were observed for Er_3_Fe_5_O_12_ samples.

The authors have a low values of the magnetic properties and the character of the hysteresis curve for the Sm_3_Fe_5_O_12_ and Pr_3_Fe_5_O_12_ samples indicated super paramagnetic behavior [21]. It is known that the total magnetic moment below 330 K is the sum of the magnetic moments of the samarium and praseodymium sublattices, respectively, and the resulting magnetic moment of the iron sublattice. Moreover, the large difference in the magnetic properties of Sm_3_Fe_5_O_12_ and Pr_3_Fe_5_O_12_ in comparison to Er_3_Fe_5_O_12_ is due to the fundamental difference in the electronic configurations of the yttrium and cerium subgroups (the cerium subgroup includes Sm, Pr, and the yttrium contains Er). This is explained by the different interactions and orientations of the spin (S) and orbital (L) magnetic moments for the materials belonging to different subgroups [32].

## 4. Conclusions

Based on the results obtained in the course of this work, the following conclusions can be drawn:

− Samples synthesized by the hydrothermal method are formed from fine-crystalline conglomerates with particle sizes from 1 to 10 microns. Chaotic morphology is observed. The porous fine-crystalline undirected structure of the samples, together with the magnetic properties of the material, can be successfully used in the manufacture of active elements of filters for industrial wastewater treatment;
− Based on the data obtained by the XRD method, peaks corresponding to the cubic structure of Sm_3_Fe_5_O_12_, Pr_3_Fe_5_O_12_, and Er_3_Fe_5_O_12_ ferrite—garnets are observed on all the presented powder diffractograms, which confirms the chemical composition of the synthesized samples. During the synthesis, the formation of residual reaction products in the form of Fe_2_O_3_ also occurs.
− The results of Raman spectroscopy confirm the chemical composition of the powders.
− The use of the AFM method allowed us to study the general nature of the structure of the entire surface of the object at small magnifications and to study in detail any area of interest at large magnifications. At the same time, there is no need to develop special sighting methods. The transition from small to large magnifications was quick and easy;
− Using scanning probe microscopy, experimental results were obtained for studying the shape and particle size of micro/nanostructured films of rare-earth ferrite-garnet, as well as the substrate material based on Al_2_O_3_

The relationship between the magnetic properties and the micro/nanocrystalline structure of ferrite-garnet powders is established.

The results obtained in this study provide a solution to a number of problems arising in the design of various devices of modern microelectronics through scientifically grounded recommendations for the synthesis of powder and film structures with optimal values of coercive force, saturation field, and magnetic effects.

The experimental study of the characteristics of the microstructure of these materials is necessary because magnetic characteristics, such as coercive force and initial magnetic permeability, are associated with structural inhomogeneities. Therefore, it is only through the directional formation of a certain micro/nanostructure of magnetic powders and films that a set of characteristics can be obtained to make them suitable for industrial use.

The results presented in this study are the initial stage of an extensive research work, the completion of which will allow for the creation of functional materials with specific properties to solve particular problems. In the future, it is planned to expand the range of materials under study, and to show how synthesis parameters (i.e., concentration and type of alkali, as well as temperature) affect the structural features of samples. The adsorption, magnetic, and sensor properties of the materials will also be presented.

In near future we intend to explore the samples synthesized by us by method of magnetic force microscopy (MFM) which combines modern technology of magnetic measurements and unique capabilities of probe microscopy. MFM is specifically relevant for the research of the magnetic properties of nanostructures—to detect quantum dimensional effects, in particular.

## Figures and Tables

**Table 1:** Chemical reagents used in the synthesis of the samples

The Name of the Reagent	Chemical Formula
Samarium Nitrate Hexahydrate (III)	Sm (NO_3_)_3_·6H_2_O
Praseodymium Nitrate Hexahydrate (III)	Pr(NO_3_)_3_·6H_2_O
Erbium Nitrate Pentahydrate (III)	Er(NO_3_)_3_·5H_2_O
Iron Nitrate Nonahydrate (III)	Fe (NO_3_)_3_·9H_2_O
Alkali	NaOH

## Data Availability

Data available in a publicly accessible repository that does not issue DOIs. Publicly available datasets were analyzed in this study. This data can be found here: The Cambridge Crystallographic Data Centre, DASH 3.3.6 www.ccdc.cam.ac.uk https://ru.qaz.wiki/wiki/Cambridge_Structural_Database (accessed on 4 April 2021). Software for Scientists Crystal Impact, Match 2 https://crystalimpact.com (accessed on 4 April 2021).
